# Marine Natural Products with Activities against Prostate Cancer: Recent Discoveries

**DOI:** 10.3390/ijms24021435

**Published:** 2023-01-11

**Authors:** Eleonora Montuori, Caroline A. C. Hyde, Francesco Crea, Jon Golding, Chiara Lauritano

**Affiliations:** 1 Department of Chemical, Biological, Pharmaceutical and Environmental Sciences, University of Messina, Viale F. Stagno d’Alcontres 31, 98166 Messina, Italy; 2 Department of Ecosustainable Marine Biotechnology, Stazione Zoologica Anton Dohrn, Via Acton 55, 80133 Napoli, Italy; 3 Cancer Research Group, School of Life Health and Chemical Sciences, The Open University, Walton Hall, Milton Keynes MK7 6AA, UK

**Keywords:** marine organisms, prostate cancer, natural products, mechanisms of action

## Abstract

Prostate cancer is the most common cancer in men, with over 52,000 new cases diagnosed every year. Diagnostics and early treatment are potentially hindered by variations in screening protocols, still largely reliant on serum levels of acid phosphatase and prostate-specific antigen, with tumour diagnosis and grading relying on histopathological examination. Current treatment interventions vary in terms of efficacy, cost and severity of side effects, and relapse can be aggressive and resistant to the current standard of care. For these reasons, the scientific community is looking for new chemotherapeutic agents. This review reports compounds and extracts derived from marine organisms as a potential source of new drugs against prostate cancer. Whilst there are several marine-derived compounds against other cancers, such as multiple myeloma, leukemia, breast and lung cancer, already available in the market, the presently collated findings show how the marine environment can be considered to hold potential as a new drug source for prostate cancer, as well. This review presents information on compounds presently in clinical trials, as well as new compounds/extracts that may enter trials in the future. We summarise information regarding mechanisms of action and active concentrations.

## 1. Introduction

**Figure 1:**
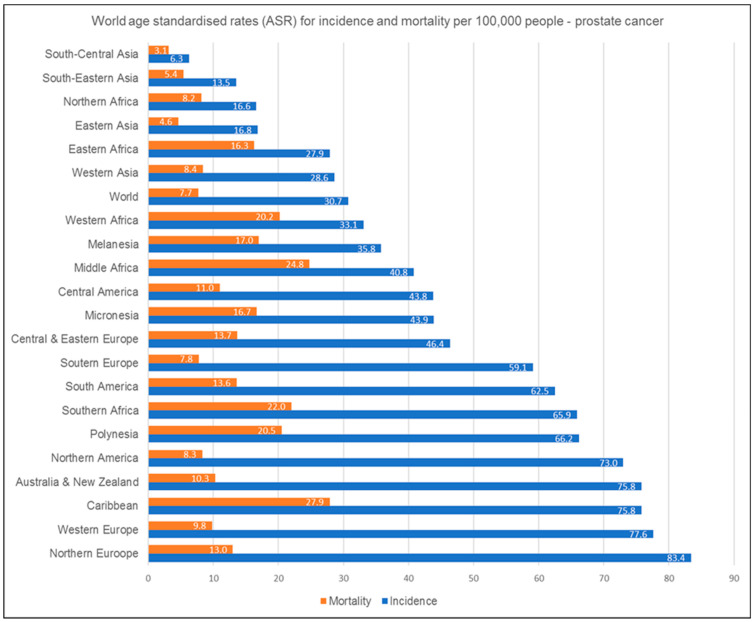
World prostate cancer incidence and mortality rates per 100,000 people. ASR stands for age-standardised rates, adapted from Globocan, 2020 [1].

Prostate cancer is the second leading cause of cancer-related death in males [1]. On a global scale, prostate cancer, along with lung and colorectal cancers, account for the majority of all cancers diagnosed in men in 2020 [1]. However, whilst the global age standardised incidence and mortality rates per 100,000 people equal 30.7 and 7.7, respectively, there is a great deal of variation in these rates on a regional level, with the majority of Asian regions featuring rates below the global average (Figure 1) [1].

In the US, prostate cancer currently accounts for 14% of newly diagnosed cancer cases, with an estimated 5.7% of cancer deaths in 2022 and a calculated 5-year survival rate for the period 2012–2018 of 96.8% [2]. Within Europe and based on data from 2020, prostate cancer accounts for 22.2% of newly diagnosed cancers in men across all ages, swiftly followed, as above, by lung and colorectal cancer, which collectively account for just over 50% of cancers [3]. In terms of mortality, prostate cancer accounts for 10% of cancer deaths in males across all ages, translating into an age-standardised incidence rate of 148.1 and an associated mortality of 38.2 per 100,000 [3]. Similarly, the 2020 data for the UK shows that prostate cancer accounts for 26.6% of newly diagnosed cancers and an associated cancer mortality of 13.9% [3]. In terms of age-standardised rates, the UK currently shows both higher incidence and mortality rates than reported for Europe, with an incidence of 186.1 and an associated mortality of 46.3 per 100,000 [3]. Overall, data from EUROCARE-5 show that there is much geographical variation within Europe, from the highest 5-year survival rates being reported at >90% for Northern Europe and the lowest around 80% in Eastern Europe [4].

Whilst tumours from distant organs can spread to the prostate, over 95% of prostate cancers develop locally as primary adenocarcinomas, with approximately 4% attributed to transitional cell carcinomas of the epithelium of the urethra or ducts [5].

Prostate cancer screening largely relies on measuring the serum levels of acid phosphatase and prostate specific antigen (PSA), even though the predictive value of this test has been questioned [6]. Diagnosis is confirmed by histopathological examination of biopsies and/or prostate membrane-specific antigen (PMSA) PET/CT scans [7], as well as tumour grading. A new “Gleason Group” grading system has been introduced to improve prognostic accuracy and to guide treatment decisions [8]. This system identifies 5 main Gleason groups: Group 1 neoplasms have the best prognosis, whilst Group 5 neoplasms have the worst prognosis. Several imaging techniques are then employed to determine the cancer stage: I (localised within the prostate gland), II/III (locally advanced), IV (metastatic). Diagnosis is often complicated by the fact that early disease stages are frequently asymptomatic, with only around 10% of patients identified during routine screening assessments, and relying on histological confirmation of disease.

Regarding treatment, a prostate cancer diagnosis often results in one or more of the following courses of action: watchful waiting/surveillance, hormonal treatment, surgical removal of the primary tumour, curative or palliative brachytherapy, cryotherapy, or curative or palliative chemotherapy. Overall, tailored treatment plans are carefully evaluated and largely driven by tumour grade and stage, circulating PSA levels and calculated risks of recurrence [9]. Stage I, low-grade tumours are treated conservatively (watchful waiting or local radiotherapy); localised tumours with more aggressive features (high grade) are surgically removed and/or treated with radiotherapy; and metastatic tumours are treated with androgen-deprivation therapy (ADT). ADT is effective because all prostatic adenocarcinomas rely on androgen receptor (AR)-dependent signalling for proliferation [10]. Upon ADT, some prostate cancers develop further genetic alterations (e.g., amplification of the AR locus) that make them “castration-resistant” (i.e., refractory to ADT) [11]. These neoplasms can still be treated with next-generation AR pathway inhibitors (e.g., enzalutamide and abiraterone) and/or with taxanes [12,13]. Approximately 20% of castration-resistant adenocarcinomas can further progress to an AR-negative neoplasm called “neuroendocrine prostate cancer” (NEPC) [14]. These highly aggressive tumours are resistant to any hormonal treatment and currently cannot be treated. The median survival time for NEPC is less than one year. Hence, there is a dire need for new therapeutic strategies for NEPC.

Whilst prostate cancers are generally seen as initially slow-growing and relatively benign tumours, treatment interventions vary in terms of their efficacy, cost and severity of side effects. Relapse, on the other hand, can be aggressive and resistant to the current standard of care. As such, the discovery of new chemotherapeutic agents as single-line treatment or in combination with radiotherapy, with both greater efficacy and preferably more favourable off-target side effect profiles, are desirable. Examples are recent studies focusing on T-type calcium channels as emerging promising therapeutic targets [15] and on the implications of long noncoding RNAs in drug resistance [16,17].

## 2. Why Marine Organisms and Which Are the Marine-Derived Drugs on the Market?

Around 70% of the planet’s surface is covered by water [18], and marine habitats have been shown to be characterized by a huge biological and chemical diversity. According to the World Register of Marine Species [19], the number of known marine species is ~241,723. In addition, several research projects focusing on the study of the diversity, distribution and abundance of marine organisms show that that the total number of marine species worldwide is much higher (e.g., the Census of Marine Life [20]), especially considering the vastness of less explored habitats, such as the deep sea and polar regions. Regarding the known marine natural products (MNPs), according to the database MarinLit, dedicated to marine natural products research [21], there are currently 39,312 articles on MNPs and 39,560 known compounds. So, why look for further MNPs? The increasing interest in MNPs is largely due to the new chemical structures available in the marine realm. In fact, 70% of structural scaffolds identified in MNPs are specific to marine organisms, with greater chemical novelty compared to their terrestrial counterparts [22,23].

**Figure 2:**
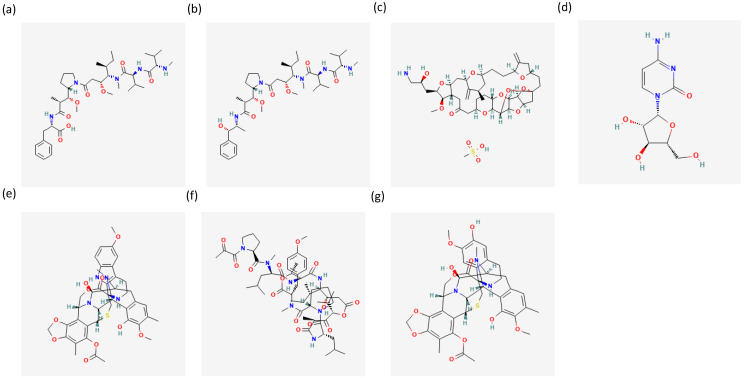
This figure shows chemical structures of (**a**) MMAF [28], (**b**) MMAE [29], (**c**) eribulin [30], (**d**) cytosine arabinoside [31], (**e**) lurbinectedin [32], (**f**) plitidepsin [33] and (**g**) trabectedin [34]. Chemical structures are retrieved from the public database PubChem [35].

A series of primary and secondary metabolites of marine organisms with potential applications for the prevention and treatment of different cancers have been reported to date [24,25,26,27]. Currently, there are 14 marine-derived drugs on the market, 9 of which are for cancer treatment. As reported in Table 1, these drugs have shown efficacy against a number of different cancer types, including multiple myelomas, leukaemias, soft tissue sarcoma and lung, breast and ovarian cancers. Regarding their chemical structures, some of these compounds are antibody-drug conjugates with monomethyl auristatin E (MMAE) or monomethyl auristatin F (MMAF); the other drugs are based on an alkaloid, macrolide, nucleoside or depsipeptide compounds (Figure 2).

**Figure 3:**
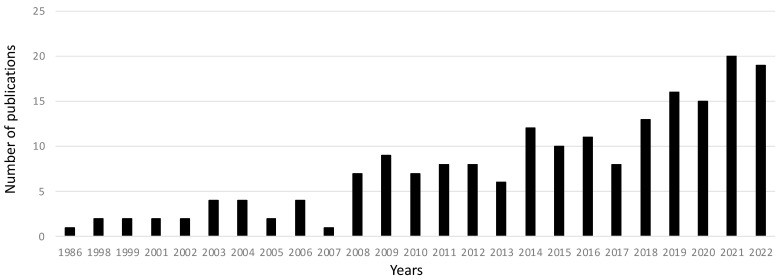
PubMed Search Results. Number of publications found by using the PubMed database search (National Library of Medicine, https://pubmed.ncbi.nlm.nih.gov/; accessed on 3 January 2023) with the keywords “prostate cancer” and “marine compounds”.

Using the search terms “prostate cancer” and “marine compounds” in PubMed, an increase in the number of publications on this topic in recent years is apparent (Figure 3). Previous reviews on marine-derived compounds with activities against prostate cancer were focused on specific compound classes, such as active peptides [37], or were focused on molecules with activities on a range of cancers [38,39], or did not include most recent findings [40]. The aim of the current review is to summarise recent discoveries of marine compounds with activities against prostate cancer, reporting, when available, the active concentrations and mechanisms of action. In addition, we highlight, where possible, those compounds that have already reached clinical trials.

## 3. Marine Microorganisms

Marine microorganisms have attracted much attention in recent years because not only do they allow drug discovery without impacting marine ecosystems, but cultivation volumes are also readily scalable in the majority of cases. In addition, mass cultivation can be supported by photobioreactors, where parameters such as light, temperature, nutrients and degree of bubbling can be modified and controlled in order to induce the production of the metabolites of interest. Bioactivities of microorganism extracts/compounds against prostate cancer cells have been reported for marine fungi, bacteria, cyanobacteria and microalgae (Table 2).

### 3.1. Fungi

Sansalvamide A is a cyclic depsipeptide, originally isolated from a marine fungus of the genus *Fusarium* found on Little San Salvador Island, Bahamas. Because N-methylation can enhance both potency and selectivity for peptides, Liu et al. [41] synthesized 12 different N-methylated sansalvamide A peptide analogues, 2 of which (compounds 5 and 11) were shown to be more potent than sansalvamide A against PC3 cells, a classical prostate cancer cell line, at 10 µM concentrations.

A number of derivatives were isolated after cultivation of the fungus *Isaria felina* KMM 4639 and subsequent fractionation of ethyl acetate extracts. They were, notably, isariketide A and isariketide B (with a hydroxyl group replacing the acetyl group in isariketide A), plus a third compound (compound 3), a derivative of isariketide A with an additional acetyl group [42]. Co-cultivation for 2 weeks with another marine fungus, *Aspergillus sulphureus*, gave rise to an additional compound, Oxirapentyn L. The cytotoxic effects of isariketide A, isariketide B, compound 3 and Oxirapentyn L were assessed on different prostate cancer cell lines (22Rv1, PC3 and LNCaP), as well as murine non-malignant splenocytes and erythrocytes. Isariketide A and compound 3 were found to be highly cytotoxic to cancer cells, whereas isariketide A showed no toxicity in normal cells until used at concentrations ≥ 100 μM. In addition, cytotoxicity was positively associated with the degree of acetyltation. Preliminary analyses of the mechanism of action of isariketide A and compound 3 suggested induction of apoptosis at 5 μM concentrations (48 h treatment). Interestingly, isariketide A and compound 3 demonstrated cytotoxicity in both hormone therapy-resistant cell lines 22Rv1 and PC3, as well as hormone-sensitive LNCaP cells, therefore presenting as promising drug substrates for drug-resistant prostate cancer therapy.

The fungus *Microsporum* sp. strain MFS-YL was collected from the surface of the marine red alga *Lomentaria catenata*, found in the Republic of Korea, and cultured for 30 days. The fermentation broth was extracted with ethyl acetate, fractionated and the pure compound physcion successfully isolated, as confirmed by nuclear magnetic resonance (NMR) and mass spectral (MS) analyses [43]. Physcion was evaluated at different concentrations (0–100 μM) and found to decrease proliferation of PC3 cells in a dose-dependent manner, with activity observed starting at 25 μM. At the molecular level, physcion induced the down-regulation of the anti-apopotoic proteins Ras, Bcl-xL and Bcl-2, the up-regulation of the pro-apoptotic protein Bax, and activation of caspases 3, 8 and 9.

SZ-685C, a marine anthraquinone isolated from the fungi of mangroves from the South China Sea [44], demonstrated cytotoxic effects in PC3 cells via pro-apoptotic activation of caspases 8 and 9, and the Akt/FOX0 pathway [44,45].

### 3.2. Bacteria

Planctomycetes phyla, one of the less studied bacterial phyla for drug discovery, have been previously shown by in silico genomic analyses to hold potential for producing many secondary metabolites. This was exploited by Calisto et al., (2019) [46], who cultivated 39 Planctomycetes strains from different habitats (including a marine water column, marine macroorganism surface, deep marine iron hydroxide deposits, brackish water and glacier ice system). All the strains were cultivated for 7 days, except one strain (named L2), which was also grown for a longer stationary phase (15 days) to evaluate the possible bioactive compound production under that condition. For these species, both organic and aqueous extracts were prepared and evaluated in both PC3 cells and the normal rat kidney epithelial cell line, NRK. Out of 39 strains, 4 were shown to possess toxicity against PC3 cells, 2 of which were selective for cancer cells, suggestive of their potential as future anticancer compounds.

Hawas et al. [47] cultured the bacterium *Streptomyces* sp. Mei37, isolated from the muddy sediment of Jade Bay on the southern German North Sea coast, and performed solid phase and ethyl acetate extractions. They identified five isoquinolinequinones, the known 3-methyl-7-(methylamino)-5,8-isoquinolinedione and four new derivatives, mansouramycin A-D, and tested them on various cancer cell lines, including DU-145 prostate cancer cells. Mansouramycin C was the most active, with IC50 values ranging from 0.24 to 1.11 µM. More recently, a secondary metabolite, named Lu01-M, was isolated from the marine actinomycetes *Streptomyces* sp., collected from marine sediments at 400 m depth in Taiwan. This compound was tested at different concentrations (0.78, 1.56, 3.125, 6.25 and 12.5 μg/mL) for 24–72 h on various prostate cancer cell lines, including PC3, DU145 and LNCaP cells [48]. Findings suggested that cytotoxicity was mediated by multiple mechanisms, such as induction of apoptosis, necroptosis, autophagy, G2/M-phase cell cycle arrest and DNA damage.

Chromopeptide A is a depsipeptide isolated from the marine sediment-derived bacterium *Chromobacterium* sp. [49]. This compound has demonstrated interesting potential for epigenetic modification by inhibiting the enzymatic activities of histone deacetylases (HDACs) 1, 2, 3 and 8 [49], known to be both correlated with poorer prognosis and to be abundantly expressed in prostate cancer. Chromopeptide A was able to suppress the proliferation of PC3, DU145 and LNCaP cells, with an IC50 of 2.43, 2.08, and 1.75 nmol/L, respectively. In addition, it increased caspase-3 activities and PARP cleavage in all three cell lines and promoted G2/M phase cell cycle arrest in PCa cells by suppressing cdc2 and cdc25C phosphorylation. Intravenous administration of chromopeptide A (1.6, 3.2 mg/kg, once per week for 18 days) in mice bearing PC3 prostate cancer xenografts significantly suppressed tumor growth [49].

The bacterial isolates 12 Y, 12 OR and 12 W from the marine sponge *Phyllospongia lamellosa* were evaluated for possible extracellular L-asparaginase production [50]. L-asparaginase is an enzyme known to have anticancer properties and is currently in use for the treatment of acute lymphoblastic leukemia, acute myeloid leukemia and non-Hodgkin’s lymphoma [51]. Different formulations are used against acute lymphoblastic leukemia, such as the native L-asparaginase from *Escherichia coli* (*E. coli*) (Elspar^®^ and its pegylated form Oncaspar^®^) and L-asparaginase from the bacterium *Erwinia chrysanthemi* (Erwinase^®^) [52]. However, considering the significant side effects, including low catalytic activity, hyperglycemia, increased liver fat and possible mild brain dysfunction, studies are focusing on alternative sources, and marine organisms present interesting new possibilities [51,53]. Partially purified L-asparaginase derived from the marine *Bacillus safensis* MK541039 was evaluated in PC3 cells for 24 h, and a concentration-dependent decrease in cell viability, with an IC50 of 38.75 IU/mL, has been reported [50].

### 3.3. Cyanobacteria

The marine cyanobacterium *Lyngbya majuscule*, collected from Papua New Guinea, produces aurilides B and C. Both compounds were tested on various cancer cell lines, and aurilide B exhibited high cytotoxicity (with GI50 less than 10 nM) against prostate cancer cells [54]. Cyclodepsipeptide lagunamide C was isolated from the cyanobacterium *L. majuscula* from the western lagoon of Pulau Hantu Besar, Singapore [55]. This compound similarly displayed cytotoxic activity against a panel of cancer cell lines, including PC3 cells, with an IC50 of 2.6 nM after 72 h. Cryptophycin 1 is a marine natural product, originally isolated from the cyanobacterium *Nostoc* sp. GSV 224 [56]. Its analogue, cryptophycin 52 (LY355703), was shown to have activity against PC3 and DU-145 (androgen-independent) and LNCaP (androgen-dependent) cells [57]. Whilst activity in PC3 cells was less pronounced, after 48 h of exposure to cryptophycin 52 (1–10 pM), LNCaP and DU-145 cells accumulated in G(2)-M cell phase and progressively acquired sub-G(0)-G(1) DNA content, suggesting apoptosis. Cryptophycin 52 induced activation of caspase-3 and caspase-7, cleavage of the caspase substrate poly (ADP-ribose) polymerase and phosphorylation of c-raf1 and bcl-2 and/or Bcl-xL. Interestingly, LNCaP cells overexpressing bcl-2 were found to be more resistant to apoptosis induction by the compound, and it was hypothesiszed that cryptophycin 52 induced apoptosis in an androgen-independent mechanism, primarily though apoptosis-associated proteins and pathways.

### 3.4. Microalgae

Finally, other anti-prostate activities from marine microorganisms were observed for compounds isolated from microalgae [58]. In particular, aqueous extracts derived from raw marine microalgal material from Canada (dried powder) were tested on PC3 and DU145 cells for 72 h at concentrations of 1, 2 and 5 mg/mL, with a reduction in cell viability only observable at concentrations of 5 mg/mL [59].

**Figure 4:**
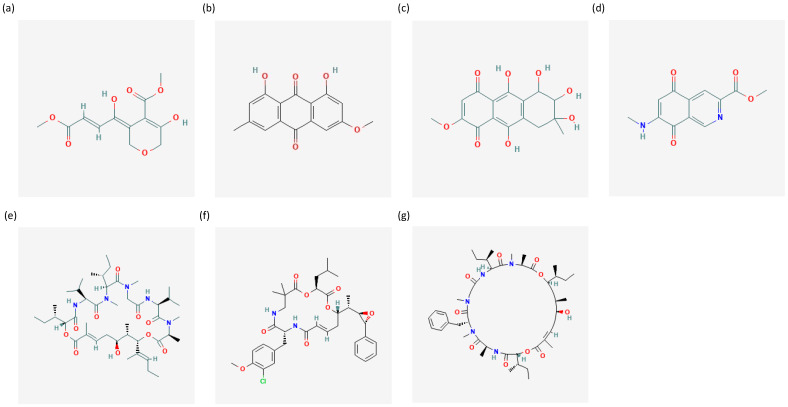
The figure shows chemical structures of (**a**) isariketide B [60], (**b**) physcion [61], (**c**) SZ-685C [62], (**d**) mansouramycin C [63], (**e**) aurilide B [64], (**f**) cryptophycin 52 (LY355703) [65], (**g**) lagunamide C [66]. Chemical structures are retrieved from the public database PubChem [35].

Chemical structures available in the public database PubChem [35] of compounds isolated from marine microorganisms with activities against prostate cancer are reported in Figure 4. Active compounds from marine microorganisms are summarised in Table 2.

**Table 2:** Compounds/extracts from marine microorganisms with activities against prostate cancer. Abbreviations: MTT for 3-(4,5-dimethylthiazol-2-yl)-2,5-diphenyltetrazolium bromide, WST-1 for water-soluble tetrazolium salt, GI50 for 50% cell growth inhibition, IC50 for half maximal inhibitory concentration, PC3, LNCaP and DU-145 for different prostate cancer cells.

Organism	Compound/Extract	Active Concentration	Assay Information	Mechanism of Action	Ref.
**Fungi**					
Originally from the genus *Fusarium*	Compounds 5 and 11 (N-methylated sansalvamide A)	10 µM	72 h exposure; cell counting; PC3 cells	-	[41]
*Isaria felina* KMM 4639	Isariketide A and compound 3	5 μM	48 h exposure; MTT assay; PC3 cells	Apoptosis induction	[42]
*Microsporum* sp. MFS-YL	Physcion	25 μM	72 h exposure; MTT assay; PC3 cells	Apoptosis induction; Downregulation of Ras, Bcl-xL, and Bcl-2 proteins and upregulation of Bax, caspases-3, 8 and 9.	[43]
Mangrove-associated fungus from South China Sea	SZ-685C	IC50 3–9.6 μM	48 h exposure; MTT assay; Annexin V assay	Apoptosis induction; Activation of caspase-8 and caspase-9	[44]
**Bacteria**					
*Chromobacterium* sp.	Chromopeptide A	IC50 2.43 ± 0.02, 2.08 ± 0.16, and 1.75 ± 0.06 nmol/L, respectively	72 h exposure; by use of sulforhodamine B; PC3, DU145 and LNCaP cells	Increased caspase-3 activity and PARP cleavage in each cell line and promoted G2/M phase arrest in PCa cells by suppressing cdc2 and cdc25C phosphorylation.	[49]
*Chromobacterium* sp.	Chromopeptide A	1.6, 3.2 mg/kg, once a week for 18 d, in mice	PC3 cells were subcutaneously inoculated	Suppression of tumor growth	[49]
39 Planctomycetes strains	Aqueous extracts	4%	72 h exposure; WST-1 assay metabolic assay; PC3 cells	-	[46]
39 Planctomycetes strains	Organic extracts	1%	72 h exposure; WST-1 assay metabolic assay; PC3 cells	-	[46]
*Streptomyces* sp.	Lu01-M	IC50 1.03 ± 0.31, 2.12 ± 0.38, and 1.27 ± 0.25 μg/mL, respectively	72 h exposure; MTT assay; PC3, DU145 and LNCaP cells	Apoptosis, necroptosis, autophagy, cell cycle arrest at the G2/M phase and DNA damage	[36]
*Bacillus safensis* MK541039	L-asparaginase	IC50 of 38.75 IU/ml	24 h exposure, by MTT assay, PC3 cells	Catalyzes the hydrolysis of L-asparagine to L-aspartic acid	[50]
*Streptomyces* sp. Mei37	Mansouramycin C and 3-methyl-7-(methylamino)-5,8-isoquinolinedione	About 0.089 µM for mansouramycin C and 0.24 to 1.11 μM for 3-methyl-7-(methylamino)-5,8-isoquinolinedione	MTT assay; DU-145 cells	-	[47]
**Cyanobacteria**					
Cyanobacterium *Lyngbya majuscule*	Aurilide B	GI50 less than 10 nM	48 h exposure, by MTT assay, panel of cells	-	[54]
Cyanobacterium *Nostoc* sp. GSV 224	Cryptophycin 52 (LY355703)	1–10 pM	48 h of exposure, by cell cucle analysis, DNA ladder formation and detection of cytoplasmic nucleosomes; PC3, LNCaP and DU-145 cells	-Apoptosis induction	[57]
Cyanobacterium *Lyngbya majuscule*	Lagunamide C	2.6 nM	72 h exposure; by MTT assay; PC3 cells	-	[55]
**Microalgae**					
Raw marine microalgal material from Canada	Aqueous extracts	5 mg/mL	72 h exposure, by MTT assay; PC3 and DU145 cells	-	[59]

## 4. Marine Macroorganisms

Similarly to marine microorganisms, invertebrates are known to produce a series of cytotoxic compounds to defend themselves from predators [67]. In the last few years, marine macroorganisms have been increasingly recognized as precious resources of natural products owing to their large reserve of secondary metabolites. The compounds eribulin, trabectedin, and monomethyl auristatin E (MMAE) are examples of three approved marine macroorganism-derived anticancer drugs (Table 1), each with their own unique mechanisms of action, explaining their ability to overcome resistance against other chemotherapeutic agents [68,69]. Recently, a number of compounds have been shown to possess activity against different prostate cancer cell lines, supporting further exploration of their suitability for the treatment of prostate cancer.

### 4.1. Sponges

In 1995, the antitumour effects of jasplakinolide, a cyclodipsipeptide produced by the marine sponge *Jaspis johnstoni*, were assessed in the human prostate cancer cell lines PC3, LNCaP and TSU-Pr1 [70]. Exposure to jasplakinolide for 48 h resulted in antiproliferative effects, with an IC50 at 65 nM, 41 nM and 170 nM in PC3, LNCaP and TSU-Pr1 cells, respectively. In particular, at 160 nM, jasplakinolide exposure for 48 h resulted in the complete growth inhibition of PC3 cells, which was accompanied by morphological changes caused by the disruption of the actin cytoskeleton [70]. In 2001, a class of alkaloid compounds containing a spermine-like substructure, named motuporamine, were isolated from the marine sponge *Xestospongia exigua.* These compounds were shown to play a role in the inhibition of invasion of basement membrane gels by PC3 cells [71]. In particular, motuporamine C was shown to inhibit cell migration and angiogenesis in monolayer cultures.

In 2006, Liu et al. demonstrated that stellettin A [72], an isomalabaricane triterpene isolated from the marine sponge *Geodia japonica*, was toxic to LNCaP cells (IC50 of 120µg/mL) and induced moderate oxidative stress, with the activation of NAPDH oxidase [72]. In 2008, a new chlorinated peptide, sintokamide A, was isolated from the sponge *Dysidea* sp. At concentrations of 5 µg/mL, sintokamide A inhibited transactivation of the N-terminus of the androgen receptor (AR) in LNCaP cells [73]. In the same year, marine-derived macrolides, latrunculin A and latrunculin B, were isolated from the Red Sea sponge *Negombata magnifica.* Both of these compounds were found to have anti-migratory activity against highly metastatic PC3M cells in a wound-healing assay and potent anti-invasive activity against PC3M cells in a matrigel assay at concentrations ranging from 50 nM to 1 μM [74]. Latrunculin A at 500 nM concentrations further decreased the disaggregation and cell migration of calcitonin-overexpressing, highly invasive PC3M-spheroids by 3-fold [74].

In 2009, (Z)-5-(4-hydroxybenzylidene)-hydantoin (phenylmethylene hydantoin) was isolated from the Red Sea sponge *Hemimycale arabica*. Phenylmethylene hydantoin showed potent anti-invasive and cytotoxic effects against PC3M cells, with an EC50 of 150 µM [75,76,77,78]. In the same year, two cyclic heptapeptides, rolloamide A and rolloamide B, isolated from the marine sponge *Eurypon laughlini*, demonstrated growth suppression of human prostate cell lines LNCap, PC3MM2, PC3 and DU145, with IC50 of 0.8 µM, 4.7 µM, 1.4 µM and 0.85 µM, respectively [79]. Also in 2009, chemical analyses of extracts from the sponge *Psammoclema* sp. led to the isolation of four new trihydroxysterols, named trihydroxysterol 1, 2, 3 and 4, related to aragusterol G. These compounds inhibited the growth of DU145 cells after 72 h [80]. In 2009, Wang et al. [81] synthesized a marine-derived makaluvamine analog, named 7-(4-fluorobenzylamino)-1,3,4,8-tetrahydropyrrolo[4,3,2-de]quinolin-8(1H)-one (FBA-TPQ). Makaluvamines are produced by marine sponges [82]. FBA-TPQ showed a dose-dependent cytotoxicity in human PC3 and LNCaP cells, with IC50 of 0.79 μM, 0.42 μM, and TRAMPC1 murine prostate cancer cells, with IC50 of 0.26 μM. Furthermore, FBA-TPQ induced apoptosis, as demonstrated by the Annexin V test and the inhibition of G2/M cell cycle transition. By contrast, IMR90-immortalized fibroblasts were relatively insensitive to FBA-TPQ (IC50 of 5.82 μM) [81].

In 2013, a trisoxazole-containing macrolide, named halichondramide, was isolated from the sponge *Chondrosia corticate*. Halichondramide showed anti-metastatic activity correlated with the down-regulation of matrix metalloproteases (MMPs) and the modulation of the expression of various biomarkers associated with metastasis, including phosphatase of regenerating liver-3 (PRL-3), proteins p85 and p110, N-cadherin, E-cadherin and matrix metallopeptidase-2 and -9 (MMP2, MMP9), at both the transcriptional and translational levels. In addition, the compound showed potent growth inhibitory activity against PC3 cells, with an IC50 value of 0.81 µM [83]. Halichondramide suppressed the expression of the potential metastatic biomarker PRL-3 in PC3 cells [84]. In 2013, the sesquiterpene ilimaquinone was isolated from the sponge *Hippospongia metachromia*. This compound promoted hypoxia-inducible factor-1 (HIF-1) and G1 phase cycle arrest in LNCaP prostate cancer cells [39,85,86].

In 2014, 20 secondary metabolites were isolated from the *Pipestela candelabra* sponge, belonging to three structural classes, including milnamide, hemiasterlin and geodiamolide. This led to the identification of three new members of the milnamide family: milnamide E, milnamide F and milnamide G, as well as a new member of the hemiasterlin family, hemiasterlin D [87]. These compounds demonstrated cytotoxic activity against PC3 cells, with IC50 values in the picomolar or submicromolar range. No cytotoxic effects were observed against the non-cancerous human neonatal foreskin fibroblast cell line NFF [87]. In 2014, a secofascaplysic acid, named 6-oxofascaplysin, was isolated from the methanolic extract of the sponge *Hyrtios sp*. Along with the known metabolites fascaplysin and reticulatate, these compounds showed cytotoxic activity against LNCaP cells, with IC50 values ranging from 0.54 to 44.9 μM [88].

In 2015, the anticancer activity of furospinosulin-1, a furanosesterpene isolated from an Indonesian marine sponge, was further assessed. It showed antiproliferative activity in DU145 cells in vitro, at 1–100 nM concentrations [89]. In hypoxia-adapted cancer cells, the mechanism of action of furospinosulin 1 was found to be mediated by the transcriptional regulators p54(nrb) and LEDGF/p75 [89,90]. In 2016, Rhizochalinin, a semi-synthetic sphingolipid-like marine compound hydrolytically derived from rhizochalin, a bioactive substance isolated from the sponge *Rhizochalina incrustata* [91], was assessed in both in vitro and in vivo studies by using prostate cancer models resistant to already approved standard therapies [92]. Rhizochalinin showed cytotoxic effects on cancer cell lines PC3, DU145, LNCaP, 22Rv1 and VCaP at low micromolar concentrations. Interestingly, the most pronounced effects were observed in 22Rv1 and VCaP cells expressing a splice variant of the androgen receptor 7 (AR-V7). AR-V7 is associated with resistance to enzalutamide and abiraterone acetate [93], and it is a constitutively active transcription factor that promotes the growth and proliferation of prostate cancer cells [94]. Regarding the mechanism of action, rhizochalinin is believed to induce caspase-dependent apoptosis, inhibit autophagy and block voltage-gated potassium channels, which are involved in metastatic spread [92,95]. In PC3 and 22Rv1 human tumor xenograft models, rhizochalinin demonstrated inhibition of both tumour growth and overall tumour mass at a dose of 1.8 mg/kg/day [92].

In 2018, the dichloromethane extract of the marine sponge *Cliona viridis* showed significant cytotoxic activity against PC3 cells, with an IC50 of 150 µg/mL. In particular, this extract decreased PC3 cell proliferation in a dose-dependent manner [96]. In 2019, the cytotoxic activity of four ceylonamide compounds, ceylonamide F, G, H and I, isolated from the Indonesian marine sponge *Sponia* sp. were evaluated in DU145 cells in 2-dimensional (2D) cell cultures [97,98]. Two of these compounds, ceylonamide G and F, showed inhibition of DU145 cell growth in 2D cultures, with an IC50 of 6.9 µM and 18.8 µM, respectively.

More recently, seven new steroids belonging to the group of gracilosulfates were isolated from the sponge *Haliclona gracilis*. After 24 h treatment, five gracilosulfate compounds, gracilosulfate A, B, D, F, and G, inhibited the expression of PSA in 22Rv1 human hormone-independent prostate cancer cells [99]. The cytotoxic activity of these compounds was also evaluated after 48 h of treatment. Whilst gracilosulfate G showed an IC50 of 64.4 ± 14.9 µM, an IC50 > 100 µM was recorded for the remaining compounds. Finally, Dyshlovoy et al. [100] highlighted the cytotoxic properties of a new compound, named spongionellol A, against prostate cancer cells, along with a known diterpene 15,16-dideoxy-15α,17β-dihydroxy-15,17-oxidospongian-16-carboxylate 15,17-diacetate, isolated from the marine sponge *Spongiella* sp. Antiproliferative activity against PCa cells was mediated by inhibition of androgen receptor (AR) signaling [101]. The AR-activated pathway is known to be essential for the survival of PCa prostate cancer cells [102]. This new mechanism of action of spongian diterpenes is indicative of their potential as anticancer agents, specifically, the observed down-regulation of the anti-apoptotic protein survivin and the up-regulation of pro-apoptotic proteins PART and caspase-3 [100].

### 4.2. Mollusks

To our knowledge, to date, only three compounds have been isolated from mollusks with proven activities against prostate cancer cell lines: kahalalide F, oculiferane and epi-obtusane. As reported below, kahalalide F is an antitumor drug under clinical investigations. It is a dipsipeptide, isolated from mollusks, that induces oncosis in human prostate cancer cells [103]. Kahalalide F showed potent cytotoxic activity on a panel of cancer cell lines, including PC3 and DU145 cells, with IC50 values of 0.07 µM and 0.28 µM, respectively. In contrast, the normal cell line IMR90 was five-fold less sensitive to kahalalide F treatment. In 2014, oculiferane and epi-obtusane, two halogenated sesquiterpenes, were isolated from *Aplysia oculifera*. These two compounds exhibited cytotoxic activity against a panel of cell lines, including PC3 cells, with IC50 values ranging from 2–8 μM [104].

### 4.3. Anthozoans

In 2010, two diterpenes, pachycladin A and pachycladin D, were isolated from the coral *Cladiella pachyclados* [105]. Both compounds showed anti-migratory and anti-invasive activities against PC3 cells after 24 h treatment at 50 µM in a wound-healing assay and in a Trevigen’s cultrex cell invasion assay [105]. Recently, pseudopterosin H was isolated from the marine coral *Pseudopterogorgia elisabethae.* Anti-neoplastic activities in PC3 cells were demonstrated both by reduction in cell viability and altering the concentration of intracellular reactive oxygen species through cytotoxic and apoptotic effects [106]. In 2018, the cytotoxic effects of the oligopeptide AAP-H isolated from the sea anemones *Anthopleura anjunae* were shown in DU145 cells [45,107]. IC50 values of 9.60 mM, 7.91 mM and 2.29 mM were reported for treatments of 24, 48 and 72 h, respectively, whilst normal fibroblast NIH-3T3 cells remained unaffected [107]. The main mechanism of action was suggested to involve the regulation of the phosphatidylinositol 3-kinase/protein kinase B/mammalian rapamycin target protein (PI3K/AKT/mTOR)-signaling pathway in order to promote apoptosis via downstream mitochondrial and death receptor pathways. In particular, AAP-H induced S-phase cell cycle arrest and induced a dose-dependent up-regulation of cleaved-caspase-3 and a down-regulation of phospho-AKT (Ser473), phospho-PI3K (p85) and phospho-mTOR (Ser2448). In an in vivo study in nude mice, AAP-H, administered at 100 and 150 mg/kg for 14 days, decreased the weight of the solid prostate tumours, with inhibition rates of 36.93 ± 3.9% in the AAP-H low-dose group and 62.22 ± 6.2% in the AAP-H high-dose group. Recently, two Sesquiterpenoids, named leptogorgin A and leptogorgin B, were isolated from the gorgonian *Leptogorgia* sp. Moderate cytotoxic properties were reported after 72 h, with selectivity against human drug-resistant prostate cancer cells 22Rv1, compared with the non-cancerous prostate cells PNT2 [108].

### 4.4. Other Macroorganisms

In 2013, Guha et al. [109] purified a Thomsen-Friedenreich disaccharide (TFD)—containing glycopeptide of molecular mass 100 kDa (named TFD_100_) from Pacific cod. TFD_100_ inhibited in vitro adhesion of androgen-independent PC3 cells to endothelial cells, angiogenesis and gal3-mediated T-cell apoptosis, and it also prevented PC3-induced metastasis in mice [109]. In 2015, Ngoan et al. [110] isolated halityloside A, halityloside B, halityloside D and culcitoside C_5_ from the starfish *Culcita novaeguineae*. These compounds were able to exhibit moderate cytotoxicity against LNCaP cells, with IC50 concentrations of 48.59 ± 2.30 mM, 39.6 ± 82.65 mM, 31.80 ± 1.59 mM and 57.0 ± 81.81 mM, respectively, assessed by determining the amount of sulforhodamine B (SRB) [110]. In a recent 2022 study, a bioactive peptide-rich salmon protein hydrolysate (SPH) showed an anticancer effect on LCNaP and PC3 cells. In particular, Bjerknes et al. showed that co-treatment of 160 µg/mL SPH with 1.0 µM bicalutamide decreased the relative colony survival of LNCaP cells from 25% to 2% after culturing for 12 days. The inhibitory effects were related to significant ferritin heavy chain 1 (FTH1) up-regulation [111].

**Figure 5:**
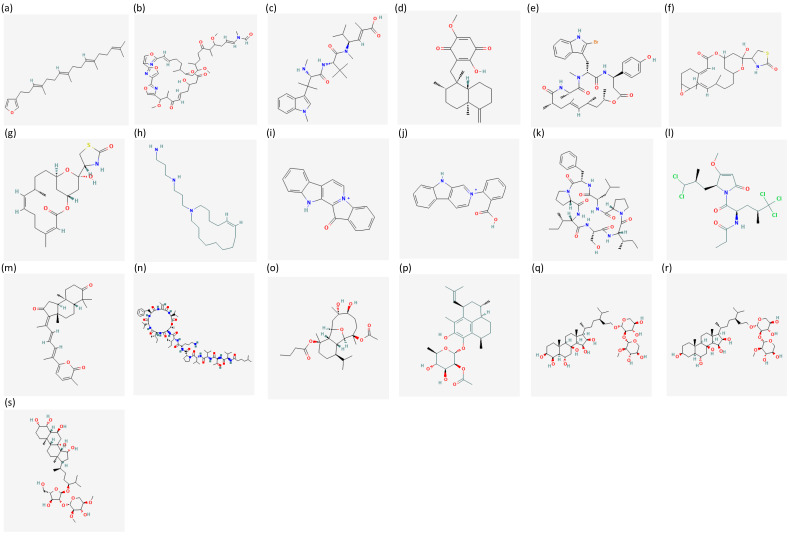
The figure shows chemical structures of (**a**) furospinosulin-1 [112], (**b**) halichondramide [113], (**c**) hemiasterlin [114], (**d**) ilimaquinone [115], (**e**) jasplakinolide [116], (**f**) latrunculins a [117], (**g**) latrunculins b [118], (**h**) motuporamine C [119], (**i**) fascaplysin [120], (**j**) reticulatate [121], (**k**) rolloamide B [122], (**l**) sintokamide A [123], (**m**) stellettin A [124], (**n**) kahalalide F [125], (**o**) pachycladin A [126], (**p**) pseudopterosin H [127], (**q**) halityloside A [128], (**r**) halityloside B [129], (**s**) halityloside D [130]. Chemical structures are retrieved from the public database PubChem [35].

Chemical structures available in the public database PubChem [35] of compounds isolated from marine macroorganisms with activities against prostate cancer are reported in Figure 5. Active compounds from marine macroorganisms are summarised in Table 3.

## 5. Marine-Derived Drugs in Clinical Trials against Prostate Cancer

There are no currently available marine drugs on the market for prostate cancer specifically, but there are a number of compounds reported in pre-clinical and early phase I/II clinical trials. A keyword search on both the ClinicalTrials.gov database, as well as the Cochrane Library, with the terms ‘prostate cancer’, ‘marine*’, ‘marine extract’ and ‘marine compound’ revealed several distinct compounds of interest as potential therapeutic candidates, in addition to a number of dietary supplementation studies making use of marine omega-3 fatty acids (not mentioned in the current study because it is focused on potential drugs). Of particular note are the pre-clinical applications of fucoidan and synthetic hemiasterlin, as well as the phase I/II studies on marine extracts in conjugate vaccines, (DSTP3086S, Tisotumab Vedotin, PSMA-ADC, Kahalalide F, fucoidan and dolastatin 10) [131,132,133].

In pre-clinical trials, the inhibitory effects of a synthetic analogue of the tripeptide hemiasterlin, a marine sponge product, were assessed in different in vitro and in vivo mouse tumour models [134]. Preliminary findings were able to demonstrate inhibition of proliferation and induction of apoptosis in both androgen-dependent and androgen-independent prostate tumours, suggesting that this may present a novel second-line treatment strategy in docetaxel-resistant prostate cancer patient populations. Furthermore, the anti-tumour and anti-angiogenic properties of fucoidan, a sulphated polysaccharide, were assessed in in vitro cell culture assays, as well as mouse xenograft models. Promising results showed an implication for the JAK-Stat3 pathway and downstream gene regulation, with direct dose-dependent inhibitory effects in cell viability, proliferation and migration [135]. These reports are in line with previous published results assessing inhibition of PI3K/Akt signalling and tumour progression in prostate cancer cells [136].

Two vaccine conjugate studies have reported the use of hemocyanin, an extract from the snail-like marine mollusk keyhole limpet. Keyhole limpet hemocyanin (KLH) has been shown to possess immune system-fortifying properties in various animals and human studies, and it has been evaluated in conjunction with MUC-2, a protein present in many cancers, including prostate cancer, and brought into solution with the immunological saponin adjuvant QS21, a tree bark extract (NCT number: NCT00698711 [137]), as well as Thomsen-Friedrich (TF) antigen, a self-antigen present on prostate cancer cells in the presence of QS21 in patients with biochemically relapsed prostate cancer [137]. Both of these vaccine studies were able to demonstrate the efficacy of KLH in breaking immunological tolerance when used in vaccine conjugates, and they were able to produce maximum IgM and IgG antibody titres against the tumour antigens used.

DSTP3086S is an antibody-drug conjugate (ADC)-targeting six-transmembrane epithelial antigens of the prostate 1 (STEAP1) conjugated to monomethylauristatin E (MMAE) in phase I trials in patients with metastatic castration-resistant prostate cancer [132]. MMAE is a synthetic derivative of dolastatin, a microtubule-destroying compound derived from the marine shell-less mollusk *Dolabella Auricularia* [138]. Tisotumab Vedotin is another ADC of tisotumab conjugated to MMAE. Tisotumab is specific for tissue factor (TF) also called thromboplastin or factor III [139]. MMAE, like all auristatins, is an anti-tubulin agent. The ADC is promoted by GenMab in partnership with Siagen Inc. [140]. PSMA-ADC is a prostate-specific membrane antigen (PSMA) ADC, again conjugated to MMAE, and it is currently in phase II clinical trials in metastatic castration-resistant prostate cancer (mCRPC) subjects who progressed following abiraterone/enzalutamide (abi/enz) therapy [141]. Kahalalide F is a depsipeptide isolated from the mollusk *Elysia rufescens* and from the algae *Bryopsis pennata*, which the mollusk feeds on (it was also hypothesized that it can be produced by a commensal microbe on the algae), in phase II trials for prostate cancer, in conjunction with patent (US20040067895A1), reporting its application against prostate cancer, in particular, androgen-independent prostate cancer [131]. Dolastatin 10 [142], originally isolated from the sea hare *Dolabella auricularia*, is the precursor compound of the reported auristatins, known to inhibit microtubules and possess apoptotic effects. Dolostatin 10 has been reported to be in phase II clinical trials in patients with hormone-refractory metastatic prostate adenocarcinoma. In addition, there is evidence for the assessment of the efficacy and safety of fucoidan in a phase I/II clinical trial study in Japan, although no linked reports of results could be located as yet (UMIN ID: UMIN000005432), with a different group of researchers reporting a role for fucoidan in raising natural killer (NK) cells in a mixed male population of tumour patients [143]. Finally, some marine-derived compounds already approved for other cancers (see Table 1) are in clinical trials for prostate cancer, such as eribulin mesylate (Identifier: NCT00337077) [144].

## 6. Discussion

Even if there are no approved marine-derived drugs against prostate cancer available yet, there are several compounds/extracts that have shown promising activities, and a number have progressed to clinical trials. The majority of those in clinical trials are antibodies directed against specific cancer cell markers conjugated with MMAE.

Regarding compounds/extracts with activities against prostate cancer not yet in clinical trials, those with the highest activity from marine microorganisms are mansouramycin C and 3-methyl-7-(methylamino)-5,8-isoquinolinedione, isolated from the marine bacterium *Streptomyces* sp. *Mei37* (active concentrations 0.089–1.11 μM); Lu01-M from marine actinomycetes *Streptomyces* sp. (IC50 1.03–1.27 μM, as shown in different prostate cancer cell lines); and chromopeptide A from the marine bacterium *Chromobacterium* sp. (IC50 1.75–2.43 μM) (Table 2). The most active compounds from macroorganisms are rolloamides A and B from the marine sponge *Eurypon laughlini* (IC50 0.8–4.7 µM)*;* helichondramide from the marine sponge *Chondrosia corticate* (IC50 0.81 μM); Kahalalide F from a mollusk (IC50 0.07–0.28 μM); and the synthetic-derived makalunamide analog FBA-TPQ (IC50 0.26–0.79 μM) (Table 3).

The major part of the studies reported in this review made use of the prostate cancer cell lines PC3, LNCaP and DU145 and compared their susceptibility to the ‘normal’ human neonatal foreskin fibroblast cell line NFF. Cytotoxicity was typically evaluated by MTT assay and induction of apoptosis assessed by Annexin V assay, with treatment periods ranging from 48–72 h. A few of the compounds were also assessed in in vivo models, such as rhizochalinin, which was administered in PC3 and 22Rv1 human tumor xenograft models at a dose of 1.8 mg/kg/day and found to inhibit tumor growth; the extract TFD_100_, which prevented PC3 metastasis in mice; and synthetic hemiasterlin and fucoidan, currently reported in clinical trials against prostate cancer.

Microorganisms are often considered more promising than macroorganisms because they can be cultivated in large volumes and quantities in eco-friendly and eco-sustainable ways and generally require shorter periods of cultivation. Furthermore, it is possible to use a One Strain Many Compounds-OSMAC approach, based on the modulation of growth parameters (light, pH, culture medium components, salinity) to improve the production of target molecules. Last, but not least, -omics studies can be a promising approach to identify the metabolic pathways responsible for the synthesis of bioactive compounds and to identify the genes responsible for their synthesis. The identified genes could be used for genetic engineering and heterologous expression experiments in order to increase the production and purity of compounds of interest.

**Figure 6:**
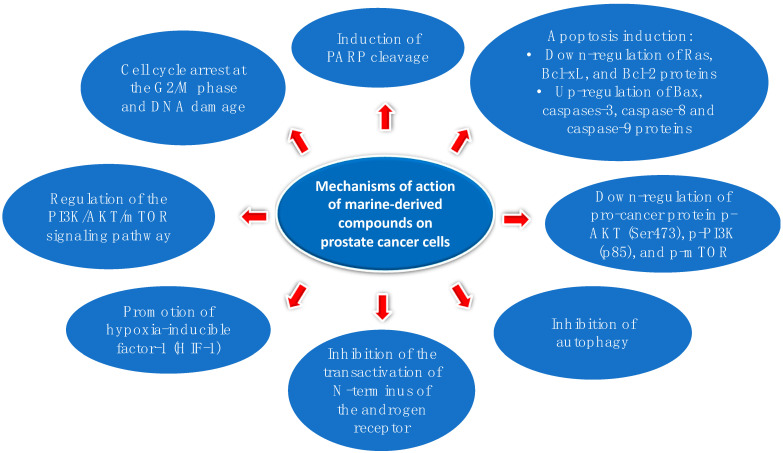
Most common mechanisms of action induced by marine-derived compounds on prostate cancer cells. PARP stands for poly ADP-ribose polimerase, PI3K for phosphatydilinositol 3-kinase, p-PI3K for phospho phosphatydilinositol 3-kinase, AKT for protein kinase B, mTOR for mammalian target of rapamycin, p-mTOR for phospho-mammalian target of rapamycin.

Overall, the data reported in the current review show that marine organisms give rise to an array of chemical structures (from peptides to complex polyketides) with a vast range of different cellular targets. However, the molecular mechanisms activated can be variable, ranging from induction of apoptosis with associated down-regulation of anti-apoptotic proteins, such as Ras, Bcl-xL and Bcl-2, and up-regulation of pro-apoptotic proteins, such as caspase-3, cleaved PARP and Bax, to the arrest of cell cycle at G2/M (summarised in Figure 6). In several cases, the mechanisms of action are still not known, and this is an exciting area for new discoveries to better characterize, optimize and advance these novel compounds into clinical trials.

## Figures and Tables

**Table 1:** Approved marine-derived drugs currently on the market for cancer therapy (as reviewed in [36]). Abbreviations: BCMA for B-cell Maturation Antigen, Ab for antibody, MMAF for monomethylauristatin F, MMAE for monomethylauristatin E and eEF1A for eukaryotic translation elongation factor 1 alpha 1. * FDA-approved. ** Australia-approved.

Species	Active Compound	Mechanism of Action	Drug Type	Drug	Cancer Type
**Mollusk/Cyanobacterium**					
Sea Hare (*Dolabella auricularia*)	Several monomethyl auristatins, derivatives of the original compound, dolastatin	Anti-tubulin agent	Ab-drug conjugate (anti-BCMA Ab conjugated to monomethyl aurostatin F (MMAF)	BLENREP^TM^ (2020) * belantamab mafodotin-blmf	Relapsed/refractory multiple myeloma
			Ab-drug conjugate (anti-Nectin 4 Ab conjugated to monomethyl auristatin E (MMAE)	PADCEV TM (2019) * enfortumab vedotin	Metastatic urothelial cancer
			Ab-drug conjugate (anti-CD79b Ab conjugated to MMAE)	Polivy ^TM^ (2019) * polatuzumab vedotin-piiq	Non-Hodgkin lymphoma, chronic lymphocytic leukaemia, lymphoma, diffuse large B-cell lymphoma
			Ab-drug conjugate (anti-CD30 Ab conjugated to MMAE)	Adcetris^®^ (2011) * brentuximab vedotin	Anaplastic large T-cell systemic malignant lymphoma, Hodgkin’s disease
**Sponges**					
*Halichondria okadai*	Synthetic derivative from original compound halichondrin B	Anti-tubulin anti-mitotic agent	Antineoplastic chemotherapeutic drug	Halaven^®^ (2010) * eribulin mesylate	Metastatic breast cancer
*Cryptotethia crypta*	Synthetic derivative from original C-nucleoside compounds	DNA alkylating agent	Alkylating chemotherapeutic drug	Cytosar-U^®^ (1969) * cytosine arabinoside (Ara-C)	Leukaemia
**Tunicates**					
*Ecteinascidia turbinata*	Derivative of the original compound ecteinascidin (trabectedin)	DNA alkylating agent	Alkylating chemotherapeutic drug	Zepzelca ^TM^ (2020) * lurbinectedin	Metastatic Small Cell Lung Cancer
*Aplidium albicans*	Synthetic derivative of original compound ascidian	Inhibitor of eEF1A	Antineoplastic chemotherapeutic drug	Aplidin^®^ ** plitidepsin	Multiple Myeloma, leukaemia, lymphoma
*Ecteinascidia turbinata*	Synthetic derived from original ecteinascidins	DNA alkylating agent	Alkylating chemotherapeutic drug	Yondelis^®^ (2015) * trabectedin	Soft tissue sarcoma and ovarian cancer

**Table 3:** Marine compounds/extracts from marine macroorganisms with activities against prostate cancer. The table reports in vitro and in vivo (in bold) available studies. Abbreviations: MTT for 3-(4,5-dimethylthiazol-2-yl)-2,5-diphenyltetrazolium bromide, WST-1 for water-soluble tetrazolium salt, GI50 for 50% cell growth inhibition, IC50 for half maximal inhibitory concentration, PCa, PC3, PC3MM2, PC3M-CT+, TSU-Pr1 LNCaP, 22Rv1 and DU-145 for different prostate cancer cells. TRAMPC1 for murine prostate cancer cell. FBA-TPQ for 7-(4-fluorobenzylamino)-1,3,4,8-tetrahydropyrrolo[4,3,2-de]quinolin-8(1H)-one. TFD_100_ for Thomsen-Friedenreich disaccharide (TFD)—containing glycopeptide of molecular mass 100 kDa. SPH for salmon protein hydrolysate.

Organism	Compound/Extract	Active Concentration	Assay Information	Mechanism of Action	Ref.
**Sponges**					
*Sponia* sp.	Ceylonamide G Ceylonamide F	IC50 of 6.9 µM IC50 of 8.8 µM	MTT assay; DU145 cells	Inhibited cell growth in 2D culture of DU145	[97]
*Cliona viridis*	Dichloromethane Extract	IC50 of 150 µg/mL	WST 1 assay; PC3 cells	Cytotoxic activity against PC3 cells; inhibits the proliferation of PC3 with in dose-dependent way	[96]
*Spongiella* sp.	15,16-Dideoxy-15α,17β-Dihydroxy-15,17-Oxidospongian-16-Carboxylate 15,17-Diacetate	IC50 2.51 ± 0.93 µM for PC3 IC50 1.52 ± 0.87 µM for DU145 IC50 1.82 ± 0.85 for LNCaP	48 h treatment MTT assay; Western blot; Annexin V assay; PCa cells	Cytotoxic effect on PCa Pro-apoptotic effect against PCa	[100]
Synthetic (originally from sponges)	FBA-TPQ	PC3 (IC50 of 0.79 μM), LNCaP (IC50 of 0.42 μM and TRAMPC1 (IC50 of 0.26 μM)	MTT assay; Annexin V test; PC3, LNCaP, TRAMPC1 cells	Dose-dependent cytotoxicity on human prostate cancer cells PC3, LNCaP and murine prostate cancer cell TRAMPC1. Induced apoptosis. Inhibited G2/M cell cycle transition	[81]
Indonesian marine sponge	Furospinosulin-1	1–100 nM.	DU145 cells	Antiproliferative activity on the prostate cancer cell line DU145 in vitro. Suppressed the growth of hypoxia-adapted cancer cells by binding to transcriptional regulators p54(nrb) and LEDGF/p75	[89]
*Haliclona gracilis*	Gracilosulfate A, Gracilosulfate B, Gracilosulfate D, Gracilosulfate F, Gracilosulfate G	IC50 of 64.4 ± 14.9 µM and IC50 > 100 µM	24–48 h of treatment; 22Rv1 cells	Inhibited the expression of prostate-specific antigen (PSA) in 22Rv1 human hormone-independent prostate cancer cells	[99]
*Chondrosia corticata*	Halichondramide	IC50 value of 0.81 µM	Sulforhodamine B (SRB) assay; wound-healing test; invasion assays; PC3 cells	Anti-proliferative activities against PC3. Anti-migratory and anti-invasive effects	[83]
*Pipestela candelabra*	Hemiasterlin D	IC50 of 2.20 nM for PC3 and 8.16 nM NFF	PC3 and NFF cells	Cytotoxic activity against PC3 and Inhibiting cells growth. No cytotoxic activity on normal cells NFF	[87]
*Hippospongia metachromia*	Ilimaquinone	-	LNCaP cells	Promoted the hypoxia-inducible factor-1 (HIF-1), induced G1 phase cycle arrest in prostate cancer cells	[85]
*Jaspis johnstoni*	Jasplakinolide	IC50 of 0.3 µM, 0.07 µM, 170 nM respectively	48 h expos ure; PC3, LNCaP and TSU-Pr1 cells	Reduction of the prostate cancer cell lines PC3, LNCaP and TSU-Pr1 growth and distruption of actin cytoskeleton	[70]
*Negombata magnofica*	Latrunculin A Latrunculin B	50 nM to 1 µM.	Wound-healing assay; Matrigel assay; PC3M-CT+ cells	Anti-migratory activity against highly metastatic human prostate cancer PC3M-CT+	[74]
*Pipestela candelabra*	Milnamide E Milnamide F Milnamide G	IC50 of 34.2 nM for PC3 and 123 nM for NFFA IC50 of 2180 nM for PC3 and 5650 for NFF IC50 of 2807 for PC3 and 30% of inhibitions for the NFF at concentration of 10 µM	PC3 and NFF cells	Cytotoxic activity against PC3 and Inhibiting cells growth. No cytotoxic activity on normal cells NFF	[71]
*Xestospongia exigua*	Motuporamine C	-	PC3 cells	Inhibited cell migration in monolayer cultures and inhibited angiogenesis in vitro	[71]
*Hyrtios* sp.	6-Oxofascaplysin Fascaplysin Reticulatate	IC50 value ranging of 0.54 to 44.9 μM.	LNCaP cells	Cytotoxic activity against the prostate cancer cell line LNCaP	[88]
*Hemimycale arabica*	Phenylmethylene Hydantoin	EC50 of 150 µM	PC3M cells	Anti-invasive and cytotoxic effect against prostate cancer cell line PC3M	[77]
*Rhizochalina incrustata*	Rhizochalinin	1.8 mg/kg/day.	PC3 and 22Rv1 human tumor xenograft models	Induced caspase-dependent apoptosis, inhibited autophagy, blocks voltage-gated potassium channels. **In in vivo experiments, rhizochalinin was administered in PC3 and 22Rv1 human tumor xenograft models at a dose of 1.8 mg/kg/day**	[91]
*Eurypon laughlini*	Rolloamide A Rolloamide B	IC50 of 0. 8 µM on LCNaP, 4.7 µM on PC3MM2, 1.4 µM PC3, 0.85 µM DU145 respectively.	MTT assay; LNCaP, PC3MM2, PC3 and DU145 cells	Growth suppression of human prostate cell lines LNCaP, PC3MM2, PC3 and DU145	[79]
*Dysidea* sp.	Sintokamide A	5 µg/mL	LNCaP cells	Inhibited the transactivation of N-terminus of the androgen receptor of prostate cancer cell LNCaP	[73]
*Geodia japonica*	Stellettin A	IC50 of 120µg/mL	48 h treatment; MTT assay; LNCaP cells	Induced cytotoxicity and moderate oxidative stress in the human prostate cancer cell line LNCaP	[72]
*Spongiella* sp.	Spongionellol A	0.964 ± 0.11 for PC3 0.936 ± 0.39 for DU145 1.02 ± 0.57 for LNCaP	48 h treatment MTT assay; Western blot; Annexin V assay; PCa cells	Cytotoxic effect on PCa Pro-apoptotic effect against PCa	[100]
*Psammoclema* sp.	Trihydroxysterols Colled Trihydroxysterol 1, Trihydroxysterol 2, Trihydroxysterol 3, Trihydroxysterol 4	GI50 13 ±1 GI50 27 ±1 GI50 27 ±1 GI50 6.7 ±1	72 h of exposure; MTT assay; DU145 cells	Inhibited the cellular growth of human prostate cancer cell DU145	[80]
**Mollusks**					
Mollusks	Kahalalide F	PC3 with a IC50 of 0.07 µM DU145 with a IC50 of 0.28 µM	PC3 and DU145 cells.	Induced oncosis, potent cytotoxic activity	[103]
Gasteropod *Aplysia oculifera*	Oculiferane And Epi-Obtusane	IG50 values ranging between 2 and 8 μM	Sulforhodamine-B (SRB) method; PC3 cells	Cytotoxic activity against PC3 cells	[104]
**Anthozoans**					
*Anthopleura anjunae*	AAP-H	IC50 of 9.605 mM, 7.910 mM, and 2.298 mM (in vitro) 150 mg/kg/day (in vivo)	24–72 h of cells treatment; DU145 cells. DU-145 xenografts for in vivo experiments	Cytotoxic effect against DU145 cells. Involved in regulation of the PI3K/AKT/mTOR signaling pathway. Induced the cell S phase arrest, a dose-dependent up-regulation of cleaved-caspase-3 apoptotic protein and a down-regulation of pro-cancer protein p-AKT (Ser473), p-PI3K (p85), and p-mTOR (Ser2448). **In in vivo experiments, APP-H reduced tumor weight and slightly increased body weight and quality of life of nude mice. AKT, PI3K, and mTOR levels were reduced in DU-145 xenografts**	[107]
*Leptogorgia* sp.	Leptogorgin A Leptogorgin B	-	72 h of treatment MTT assay; 22Rv1 cells	Moderate cytotoxicity of both compounds against 22Rv1 human drug-resistant prostate cancer cells	[108]
*Cladiella pachyclados*	Pachycladin A Pachycladin D	50 µM	24 h treatment; Wound-healing assay; MTT assay; PC3 cells	Anti-migratory and anti-invasive activities against the PC3 cells	[108]
*Pseudopterogorgia elisabethae*	Pseudopterosin H	-	MTT assay; PC3 cells	Anti-neoplastic activity in the PC3 cells	[106]
**Other Macroorganisms**					
Starfish *Culcita novaeguineae*	Halityloside A, Halityloside B, Halityloside D Culcitoside C5	LNCaP with IC50 = 48.59 ± 2.30 mM, IC50 = 39.6 ± 82.65; IC50 = 31.80 ± 1.59, IC50 = 57.0 ± 81.81	sulforhodamine B (SRB) assay; LNCaP	Moderate cytotoxicity against the human prostate cell line LNCaP	[110]
Salmon	Sph	160 µg/mL	LCNaP and PC3 cells	Anticancer effect on LCNaP and PC3 cells Decreased LNCaP cells’ relative colony survival from 25% to 2%	[111]
Pacific cod	Tfd_100_	-	PC3 cells. PC3-induced metastasis in mice	Inhibited in vitro adhesion of androgen-independent prostate cancer cells PC3 to endothelial cells, angiogenesis, and gal3-mediated T-cell apoptosis and also **prevented PC3-induced metastasis in mice**	[109]

## Data Availability

Not applicable.
